# Dynamics of Polymer Molecules with Sacrificial Bond and Hidden Length Systems: Towards a Physically-Based Mesoscopic Constitutive Law

**DOI:** 10.1371/journal.pone.0056118

**Published:** 2013-04-02

**Authors:** Ahmed E. Elbanna, Jean M. Carlson

**Affiliations:** 1 Department of Physics, University of California Santa Barbara, Santa Barbara, California, United States of America; 2 Department of Civil and Environmental Engineering, University of Illinois at Urbana- Champaign, Urbana, Illinois, United States of America; 3 Department of Physics, University of California Santa Barbara, Santa Barbara, California, United States of America; University of Akron, United States of America

## Abstract

We investigate the entropic force-elongation behavior of a polymer chain in the presence of the sacrificial bond and hidden length (SBHL) system observed experimentally in many biomaterials. We show that in most cases the SBHL system leads to a significant increase in toughness. However, the presence of a large number of bonds or relatively strong bonds in the SBHL system can reduce the net gain in toughness. We also incorporate the polymer model into a network of polymers with random properties (e.g., contour length, number and strength of sacrificial bonds, length of hidden loops). This allows us to derive a physically-based mesoscopic force-displacement law that governs the collective behavior.

## Introduction

Entropy based energy dissipation mechanisms were shown to play an important role in the dynamics of many physical systems. These include granular materials [1], glassy materials [2], thin film lubricants [3], and hard spheres [4]. Laws of statistical thermodynamics provide a framework for tracking the evolution of entropy and energy flow across different scales in these systems enabling a physically based description of the constitutive response [5]–[7]. In this paper, we use this framework to construct the force extension law for bone deformation at the scale of mineralized collagen fibrils (∼1 µm).

The mineralized collaged fibrils constitute the building blocks of the bone hierarchical structure at the sub micrometer scale. Studies on bone structure show that it is a hierarchical composite of collagen and hydroxyapatite [8]–[9] with mechanisms to resist fracture at different size scales [10]. These size scales relate to the characteristic structural dimensions in bone, which vary from twisted peptide chains at the nanometer scale to the (secondary) osteon (haversian) structures, which are several hundred micrometers in size. The hierarchical structure at the intermediate scales includes (i) hydroxyapatite-impregnated twisted collagen fibrils at the scale of tens of nanometers; (ii) collagen fibers that are typically a micrometer in diameter and (iii) the lamellar structure of collagen fibers at several micrometer dimensions. It is the simultaneous operation of the fracture-resistance mechanisms at these various length scales that provides bone with its enduring strength and toughness.

**Figure 1:**
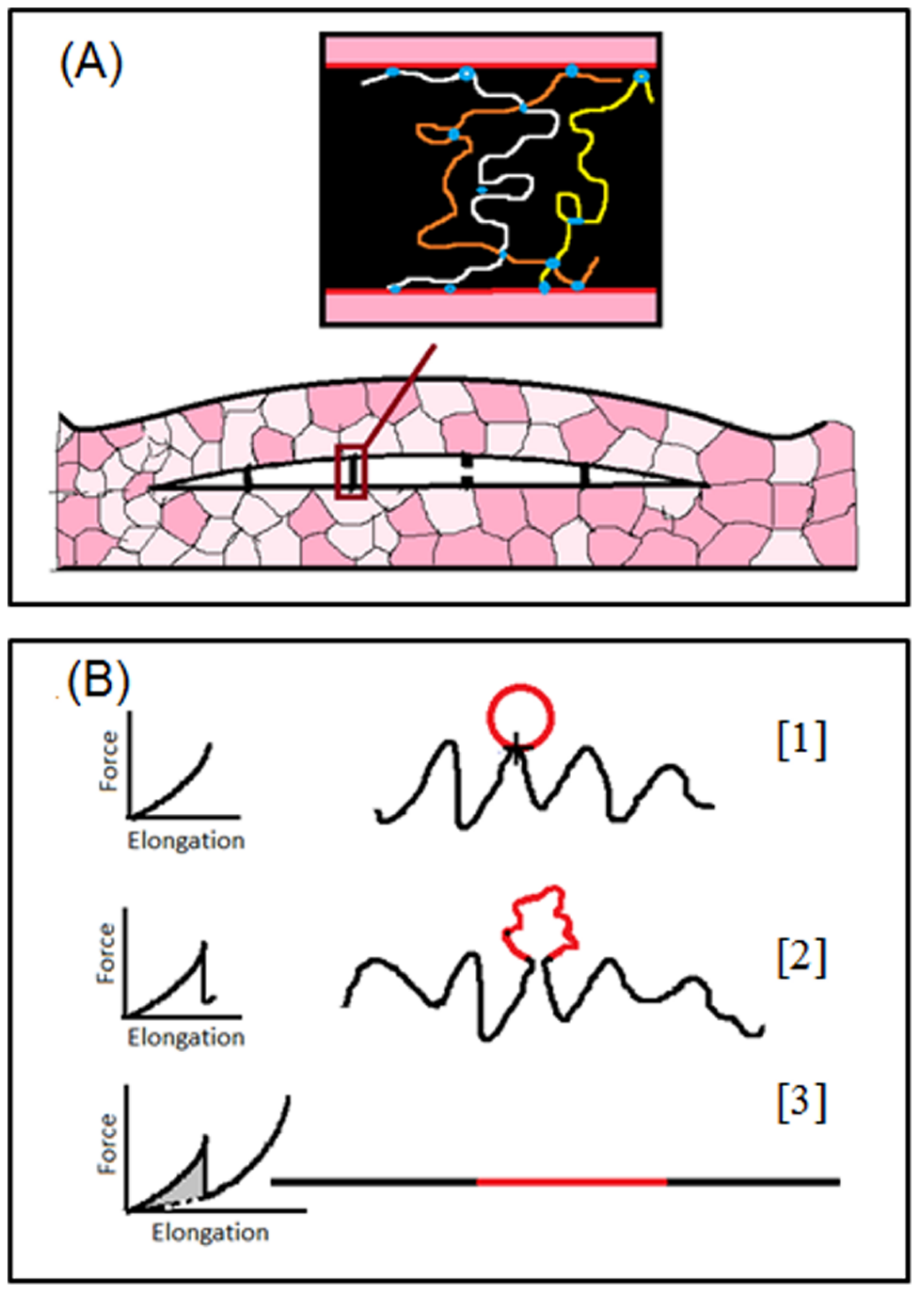
The structure and the basic operation principles of the SBHL system. (A) Hypothesized structures of assembled glue molecules, with sacrificial bonds (represented by blue circles) along the backbone of the polymer molecules resisting rupture of the molecules as they are stretched during microcracking. There are three types of bonds: (i) bonds within the polymer chain, (ii) bonds between different chains, and (iii) bonds connecting the chains to the backbone. (Adapted from Figure 3 in Fantner *et* *al.* Nature Materials 2005; 4:612–616.) (B) Schematic drawing of the basic principles of the sacrificial bond-hidden length mechanism. [1] Before a sacrificial bond is broken, only the black length of the molecule contributes to the entropic configurations and to the force with which the molecule resists stretching. The red length of the molecule is hidden from the force by the sacrificial bond. [2] When the bond fracture threshold is reached, the bond breaks and the whole length of the polymer (black plus red) contributes to its entropy. The force supported by the polymer molecule abruptly drops in response to this sudden increase in entropy. [3] As the polymer molecule is further stretched the force supported by it increases. This continues until the molecule becomes straight and breaks. The grey area represents the extra work done in stretching a polymer with SBHL system relative to a polymer of the same length but without the SBHL system.

Here, we focus on the mechanics of deformation at the scale of the mineralized collagen fibrils. Atomic Force Microscope (AFM) experiments done at this scale [11]–[12] showed that the fibrils are interconnected by a special type of glue that is composed of polymer chains with sacrificial bonds and hidden length (SBHL) systems. The basic structure and operation mechanism of the SBHL system are shown in Fig. 1. The assembled glue molecule (insert in Fig. 1a) may include more than one polymer chain with sacrificial bonds forming within the chain itself, crosslinking the different chains and connecting the chains to the collagen fibrils. The large scale separation of the collagen fibrils is resisted by an array of parallel gel molecules as shown in Fig. 1a. We use the worm-like chain model [13]–[15] (WLC) to describe the mechanical response of the glue molecules and to identify the relationship between the SBHL system internal variables and macroscopic observables such as toughness and strength. We also examine the mechanical response of the array of parallel molecules.

The operation of the SBHL system depends on the state of the sacrificial bond. As long as the bond is intact, it shields parts of the polymer length from contributing to the end-to-end distance. This corresponds to a reduction in the chain entropy (the possible number of configurations resulting in the same end-to-end distance) and a corresponding increase in the initial stiffness of the polymer chain. The increased stiffness means that a larger force is required to extend the polymer chain any finite distance, compared to that required in the case of a polymer with no SBHL system. After the sacrificial bond is broken, the shielded loop unfolds and significant energy is dissipated in reducing the chain entropy as it straightens out. Atomic Force Microscope (AFM) indentation [11], pulling [11], and imaging [16] experiments support this understanding. Moreover, these experimental results suggest that SBHL system, found in gel molecules binding the mineralized collagen fibrils together, may play a substantial mechanical role in bone [12], [17]–[19]. Order of magnitude calculations show that less than 1% by weight of this “glue” can have profound effects on the fracture resistance [12]. This challenges the conventional belief that collagen fibrils and mineral plates are the only components of bone with a strengthening mechanical role [20].

Interestingly, the SBHL system is not limited to bone. Rather, it is widespread in many remarkably tough nano-composite biomaterials such as abalone shell and diatoms [21]-[27]. Abalone shell, for example, is 97% crystalline calcium carbonate plates by weight, but is 3,000 times more fracture resistant than pure calcium carbonate [21]–[22]. The other 3% is an interstitial organic matrix, which contains extremely efficient “glue” that incorporates the SBHL system [17]. Adhesive nanofibers from live diatoms exhibit the signature of modular proteins and they show remarkable toughness [27]. The saw-tooth behavior in their force extension response corresponds to unfolding and detachment events similar to the SBHL systems mechanism [27]. A similar behavior has been reported for parallel polyprotein dimers [28] and spider capture silk [29]. A more complete understanding of the SBHL system mechanics may be useful in developing innovative therapeutic procedures as well as designing bio-inspired composite materials with improved strength and toughness.

This paper is organized as follows. In Section II we use the worm like chain model to describe the mechanics of a polymer with the SBHL system and compare the model predictions with AFM experimental observations. In Section III we investigate the influence of the SBHL internal variables, such as the number of bonds and the strength of bonds, on the macroscopic observables such as the increase in toughness. In Section IV we use the polymer force law discussed in Section II in a model of an ensemble of parallel polymers to predict the collective force-elongation behavior at mesoscopic scales. We also examine the effect of the polymer density on the collective behavior. Finally we summarize our conclusions and discuss future directions in Section V.

## Review of The Basic Mechanical Properties of Polymers with Sacrificial Bonds and Hidden Length

Polymer elasticity is essentially entropic [30]–[31]. The relationship between the chain force (*f*) and its end-to-end distance (*x*) is controlled by the multiplicity of the number of configurations the chain can take consistent with this given end-to-end distance. The higher the multiplicity, the higher the chain entropy, and the lower the force it can support. As the polymer chain is stretched, its entropy decreases and higher forces are required corresponding to a given end-to-end distance. The end-to-end distance *x* cannot exceed the contour length of the polymer chain (*L*_c_). (They coincide only when the polymer is completely straight).

The presence of the sacrificial bonds makes the length of the polymer available for entropic fluctuations smaller than the contour length (the difference is the total length of the hidden loops). In this paper, we assume that the bonds break according to a threshold fracture criterion. This is a reasonable assumption in the limit of slow pulling rates [32]. Once a bond breaks, the length of the polymer available for entropic fluctuations increases by an amount equal to the length of the freed loop. This instantaneously increases the chain entropy and leads to an abrupt drop in the force supported by the polymer molecule. With further stretching, the chain entropy decreases and the force in the chain increases.

To quantitatively describe the force-elongation response we use the worm like chain model. We show in Supporting Information Part A in File S1 that this represents a good fit for the experimental data available in the literature on bone glue [11] in the limit of large extension (Fig. S1 in File S1). The force extension relationship is given by:
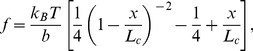
(1)where
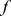
is the force, 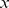
is the end-to-end distance, 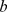
is the persistence length, 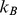
is Boltzmann' constant and 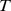
is the temperature. In the presence of sacrificial bonds, the contour length in Eqn. (1) is replaced by the available length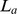. The available length is the total length of the polymer segments that can contribute to entropic elasticity. The contour and the available lengths are related by:
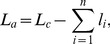
(2)where
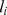
is the length of loop i shielded by the sacrificial bond i and 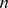
is the number of sacrificial bonds that are not broken yet. Accordingly, Eqn. (1) takes the form:



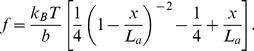
(3)In this section we review the key features describing the force extension curves observed in AFM experiments on bone glue [11] and discuss their mechanical implications. We follow a computational procedure similar to the one used by Fantner et al., 2006b [19]. In carrying out the numerical simulations, we keep track of the available length. Each time a sacrificial bond is broken, the available length increases by the length of the loop segment that is freed after the bond breakage.

We assume that a bond breaks when it is subjected to a force that exceeds a threshold value taken to be the bond strength. From the AFM experiments, the bond strength is estimated to be of the order of few 100 pN [33]. Several factors can affect this estimate including the bond chemical composition, its interaction with the surrounding solution, and other environmental and loading conditions such as the thermodynamic temperature and the pulling rate. Moreover, a given hidden loop may be shielded by more than one bond. Models based on the transition state theory can be used to account for some of these factors. They have been used to describe unfolding of polymer molecules [34] and strength of molecular adhesion bonds [32].

In this paper we do not account for such details explicitly. Instead, we will make the assumption that the bond strength is a random variable drawn from a uniform distribution between 1 pN and 1 nN (to account roughly for the different sources of variability). This should be a good approximation within the quasistatic loading limit we are considering here. The current framework can be extended to account for the effects of higher loading rates where the details of bond dynamics become significant.

**Figure 2:**
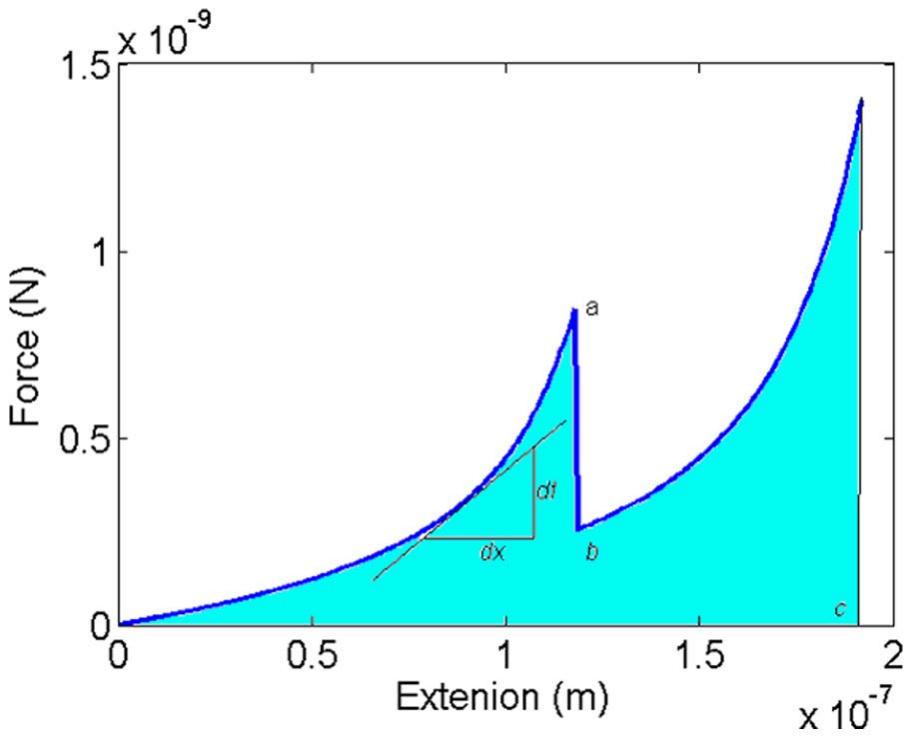
The force-extension behavior of an idealized polymer chain with a single sacrificial bond. The grey-filled area represents the work done in stretching the polymer to its fullest. This is equal to the polymer toughness. Point *a*, corresponding to the first force peak, represents the strength of the sacrificial bond. Point *b* represents the force supported by the chain just after the bond has broken. Point *c* represents the maximum extension to which the polymer chain can be stretched. This is the measure of the chain ductility. The stiffness of the chain at any given displacement is given by the slope of the straight line tangential to the force-extension curve at that displacement (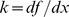).

An example of the force-extension behavior generated by Eqn. (2) for a polymer with a single sacrificial bond is shown in Fig. 2. The figure also defines several key concepts including chain stiffness, ductility, and toughness.

The chain stiffness indicates the ability of the chain to resist deformation. It is given by the slope of the tangent to force-extension curve. By differentiating Eqn. (2) with respect to *x*, we obtain the following expression for the chain stiffness:
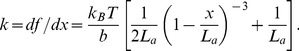
(4)


That is (i) the chain stiffness decreases as the available length increases, and (ii) the chain stiffness increases as the chain extension *x* increases. The presence of a sacrificial bond reduces the available length and hence increases the chain stiffness, making the chain stronger at smaller extensions. The saw tooth shape in Fig. 2 is characteristic of sacrificial bond breakage. The peak 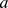 corresponds to the bond strength. The magnitude of the force drop 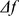 is readily computed as:
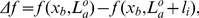
(5)where 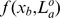 and 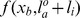 are the forces in the chain (Eqn. (2)) just before and after the breakage of the sacrificial bond respectively,
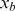
is the displacement at which the bond breaks,
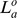
is the available length before the sacrificial bond breakage and 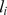 is the length of the loop freed by the breakage of the sacrificial bond *i*.

Two other important concepts, ductility and toughness, are introduced in Fig. 2. The ductility of the polymer is indicative of its ability to deform and stretch without breakage. It is measured by the maximum end-to-end distance the polymer can be stretched to before its failure or detachment from the back bone. The end-to-end distance is always less than or equal to the contour length (the equality holds only when the polymer chain becomes fully straight). Hence, the ductility of the chain cannot exceed its contour length. On the other hand, the toughness represents the work done in stretching the polymer to its maximum end-to-end distance. It is measured by the area under the force-extension line (the hatched area).

**Figure 3:**
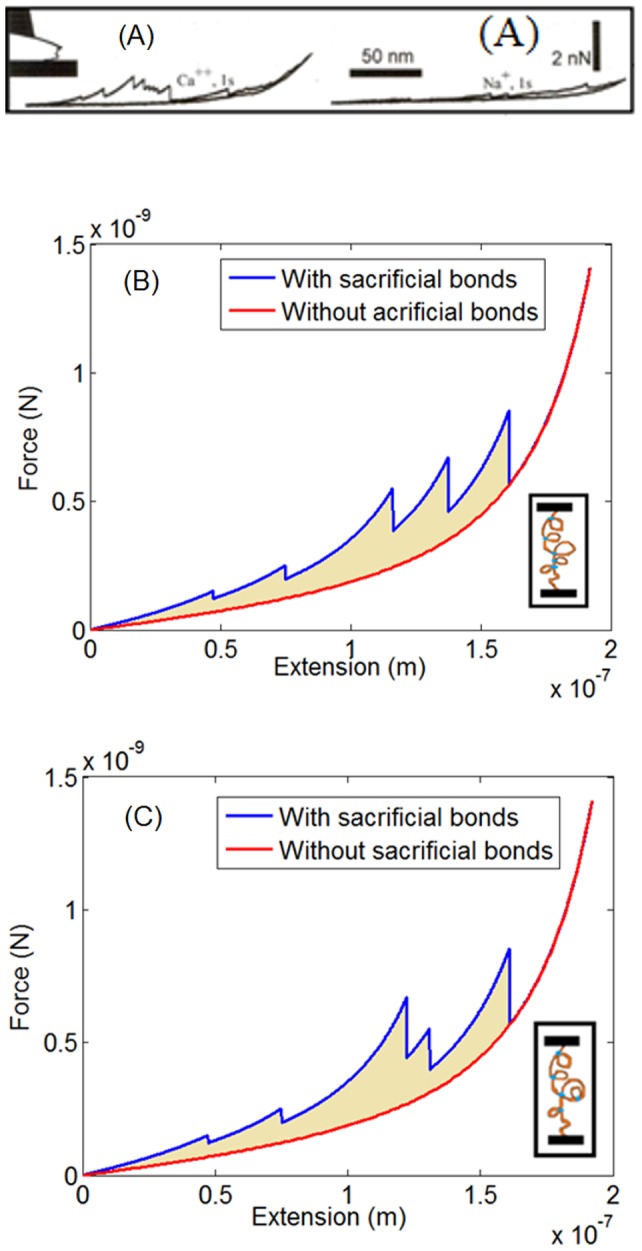
Experimental and numerical results for the force-extension response of polymer chains with SBHL system. (a) The force-extension response typically observed in AFM experiments (Reprinted with permission from [33]), (b) The force-extension response generated by the WLC model Eqn. (2) showing force peaks that are monotonically increasing (Case 1). In this case the weakest sacrificial bond breaks first. (c) The force-extension response generated by the WLC model Eqn. (2) showing force peaks that are non-monotonic (Case 2). This is consistent with the AFM experimental results (shown in Fig. 3(a)). A possible mechanism is that the weaker bond (bond no. 4 from the top) is shielded within a stronger bond loop (bond no.3 from top) and will become exposed only if the third bond breaks and its attached loop unfolds. In both Figs. 3(b) and 3(c), the blue lower line represents a polymer chain with the same contour and persistence length but with no SBHL system. The filled area between the lower blue and upper red lines represents the increase in toughness due to the presence of the SBHL system. (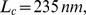 *b = *0.02 nm. It is more probable in these experiments that a single connection between the fibrils consist of a number of polymers interconnected with each other and pulled simultaneously. In this case, our model could be understood as being representative of the equivalent polymer chain with the same peak force and maximum extension).

Figure 3(a) shows an example force-elongation curve from an AFM experiment [11]. In these experiments, two pieces of the fibril plates placed in a solution are used. One piece is glued to the AFM cantilever tip and the other one is kept fixed at the base. The two pieces are connected together by the polymeric gel filaments. The pieces can be pressed together and then pulled apart to simulate the molecular interactions that occur during the separation of mineralized fibrils within bone. Experiments can be carried out either under displacement-controlled conditions or force-controlled conditions. Figure 3a shows an example from a displacement- controlled AFM experiment. After the two pieces of bone are put in contact, a force is required to separate them. The force as a function of distance during the separation phase is given by the upper line in Fig. 3(a) which consists of a number of smooth increases of the force interrupted by sudden drops. Each force drop corresponds to the breakage of a sacrificial bond or the detachment of a polymer chain from the backbone. The maximum force reported is typically of the order of nanonewtons and the maximum extension is of the order of hundreds of nanometers. As the two ends approach each other, the force in the chain relaxes and the response is described by the smoother lower line in Fig. 3(a). (No bond breakage or polymer detachment occurs during retraction).

Figures 3(b) and 3(c) illustrate our corresponding numerical results for the model when multiple sacrificial bonds and hidden loops are included. The arrangement of the force peaks in the plots depends on the topology of the polymer chain (shown to the right of the plots). In particular, we identify two cases:

Case 1 illustrates the response of a polymer with the SBHL system in which the force peaks are monotonically increasing (Fig. 3(b)). This occurs when each sacrificial bond shields only one hidden loop. With the strength of sacrificial bonds drawn from a random distribution, the weakest sacrificial bonds in this case breaks first.

Case 2 illustrates a situation in which the force peaks are not arranged monotonically (Fig. 3(c)). This latter case is more consistent with AFM experiment (Fig. 3(a)). Possible mechanisms for this non-monotonic variability in the force peaks within the force-elongation response include: (i) weak bonds contained within hidden loops [shown here] and (ii) more than one polymer chain is pulled simultaneously. We will further discuss the case of having multiple parallel connections between the fibrils in Section IV.

The discussion in this section reveals the mechanical role of the SBHL system which improves both the polymer stiffness and toughness. This leads to two key contributions to the intrinsic mechanical response of the bio-material in which the SBHL system exists:

1. Increased resistance to crack initiation: The strength of the polymer chain with sacrificial bonds is always higher at low and intermediate values of displacement. Hence, larger forces will be required to initiate a tear or increase a crack opening in the presence of the SBHL system.
2. Improved energy dissipation: The SBHL system increases polymer toughness. Tougher materials are more ductile and more resistant to brittle (catastrophic) failure. This is particularly important in cases of impact loading where the presence of sacrificial bonds will facilitate the dissipation of the impact energy in inelastic deformation and will reduce the probability of bone breakage.

## Limitations of The Sacrificial Bonds and Hidden Length System

In this section, we investigate the relationship between the SBHL system internal variables (bond strength and density) and the macroscopic response of the polymer chain in terms of its strength, ductility, and toughness. These factors are expected to play a key role in designing bio-inspired composite materials and in developing therapeutic interventions for improving bone health.

To investigate the effect of the number of sacrificial bonds (i.e. bond density per polymer) on the polymer toughness, we consider a polymer chain with a given contour length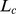 and *n* sacrificial bonds. Each bond hides a loop with length *l*. For the purpose of this estimate, we consider an idealized case in which 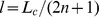
(i.e. all loops have the same length). The available length of the polymer is then readily calculated as
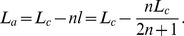
(6)


**Figure 4:**
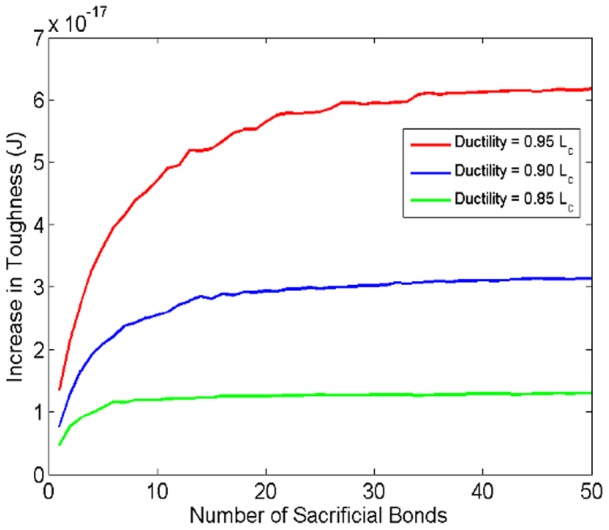
Effect of varying the number of sacrificial bonds on the increase in toughness of the polymer chain. Different curves correspond to different ductility values.

The increase in toughness is the difference in the work done to extend the polymer chain with and without the sacrificial bonds. We calculate the increase in toughness for different values of *n*. The results are shown in Fig. 4 (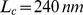
and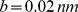).

In generating the numerical results, each point represents the average of 5000 realizations with randomly varying bonds strength. As the number of bonds increases there is a large increase in the toughness after which the curve eventually levels off.

One of the factors affecting the number of sacrificial bonds is the type of the solution in which the polymer is placed. Negative charges on the chains can combine with free cations in the solution to form sacrificial bonds [35]. Fig. 3a suggests that during the separation of fibrils, more energy is dissipated when filaments are pulled in Ca^2+^ solutions than in Na^+^ solutions. A possible explanation for this difference, in the light of the curves in Fig. 4, is that more sacrificial bonds are formed in the former (with the divalent calcium ion in the solution combining with two negative ions on the polymer backbone) than in the latter enabling a larger increase in toughness.

The asymptotic results represented by the curves in Fig. 4 can be understood from the continuum limit of the SBHL system chain as the number of bonds becomes large. For a fixed contour length, as the number of sacrificial bonds increases, the initial value of the available length approaches
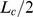
asymptotically (Eqn. (6)).In this limit, the instantaneous stiffness of the chain varies weakly with the breakage of sacrificial bonds (Supporting Information Part B in File S2) and the force-extension curves become indistinguishable from each other (Fig. S2 in File S2). Since toughness is measured by the area under the force-extension curve, this implies that there will be little or no change in toughness due to the change in the number of bonds.

The number of bonds at which the increase in toughness levels off depends on the ductility of the polymer chains. As the ductility increases, the increase in toughness for each additional (extra) bond decreases at a smaller rate. The ductility depends on the strength of the bonds connecting the chain to the collagen fibrils as we shortly discuss.

Another important control parameter for the SBHL system is the strength of the sacrificial bonds. Two types of sacrificial bonds are considered: sacrificial bonds within the polymer chain (internal bonds) and sacrificial bonds connecting the polymer to the collagen fibrils (end bonds). This distinction between the two types of bonds is important as they can be structurally different and hence can have different values of strength.

In order for the SBHL system to enhance the polymer toughness, the strength of both types of sacrificial bonds must be such that the polymer can extend to its fullest length. This implies that the strength of end bonds should be, at least, larger than the strength of all the internal sacrificial bonds. This can be explained as follows.

The force in the polymer chain (Eqn. (3)) depends on the 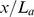
ratio (where *x* is the end-to-end distance and 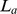 is the available length). Sacrificial bonds reduce the available length and hence higher forces develop in the chain at smaller extensions. If the internal bond is stronger than the polymer, the polymer breaks first. On the other hand, if the end bonds are weaker than the polymer, this leads to the premature detachment of the polymer chain from the collagen fibrils (Section IV). In both cases, the system fails before the polymer achieves its full extension (corresponding to its contour length). Accordingly, both the ductility and toughness are reduced.

Efforts to enhance the bone mechanical response, whether by stimulating the bone production of or by injecting the bone with gel molecules, must account for these effects. Our results suggest density of internal bonds and constraints on the strength of the internal and end bonds will be factors affecting the SBHL system performance.

## A Mesoscopic Cohesive Law for The Polymeric System

AFM experiments [12] suggest that the force supported by a fibril is of the order of a few micronewtons whereas the strength of the single polymer is of the order of several hundred piconewtons to a few nanonewtons. This means that on average 1000 polymers per fibril are required, in a healthy individual, to assure the full transfer of the force by the fibril plate.

We incorporate this in our models of the mechanical response by analyzing a group of polymer chains placed parallel to each other and constrained by the same displacement-controlled loading conditions considered previously. This setup is relevant to a wide range of applications. For example, the mechanical signature of modular proteins in diatoms [27] and parallel polyprotein dimers [28] have been previously identified in AFM experiments. Another example is quasi-brittle cracking in crystalline solids. There, the crack proceeds by breaking arrays atomic bonds intersecting with its path [36].

For the purpose of the current investigation, we consider the contour length of different polymer chains as an additional variable that is drawn from a uniform random distribution. The length of a single polymer is typically 200–350 nm. In reality, however, polymers connect with each other to form chains of much longer length enabling end-to-end extension of the order of micrometers. We assume that the total length of the chain is a random variable with a maximum value of 5 µm.

The strength of sacrificial bonds is drawn from the same random variable distribution used in Sections II and III. Each individual polymer chain deforms according to the worm like chain model (Eqn. (3)). We investigate the collective behavior of the group of polymer chains represented by the overall force-displacement response and how this response changes as a function of the number of polymer chains (*N_p_*).

At any given instant of time, all surviving polymers are constrained by the same end-to-end distance. This is achieved by envisioning a loading apparatus of infinite rigidity. In this limit, the stress disturbance, resulting from the detachment of individual polymers, equilibrates instantaneously and does not affect the force distribution on neighboring polymers.

**Figure 5:**
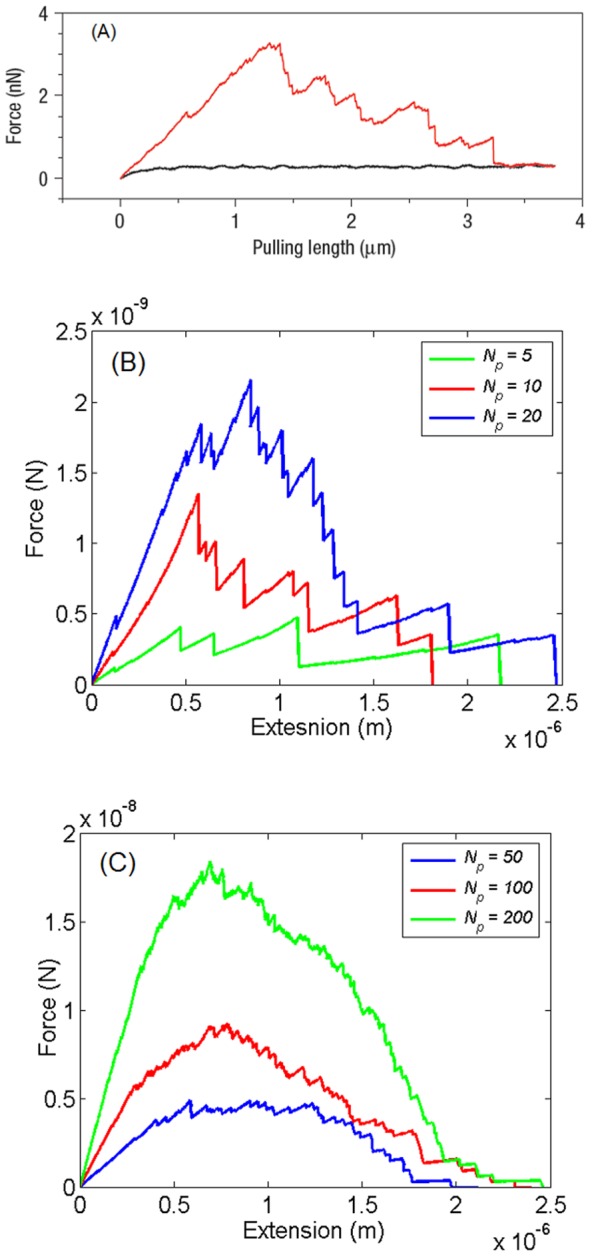
The force-extension response for different number of polymers under controlled displacement loading conditions. (a) Representative pulling curve observed in AFM experiments on dentin molecules (reprinted with permission from [35]) (b) Representative curves illustrating force-extension response for 5, 10 and, 20 polymers. The smaller force drops correspond in most cases to the breakage of sacrificial bonds while the larger force drops typically represent the detachment of polymer chains. The curve shows similar features to the plot in (a) suggesting that only a few chains are being pulled simultaneously during the AFM experiment. (c) The force extension plot for the polymeric gel when the number of polymer chains is large. The curves look much smoother than the curves in (b).

The force-extension response for different number of polymer chains is shown in Fig. 5. A representative pulling curve observed in AFM experiments on dintin molecules [35] is illustrated in Fig. 5a The case of small number (*N_p_* = 5, 10, 20) is shown in Fig. 5(b) while that for the large number (*N_p_* = 50, 100, 200) is shown in Fig. 5(c).

For small number of chains (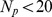) the discrete effects of sacrificial bond breakage and polymer detachment are pronounced. In this range, the results are intrinsically stochastic and sample dependent. These features have been observed in AFM experiments on blobs of dentin molecules (Fig. 5a) suggesting that a small number of chains (∼10 molecules) is being pulled in such experiments. On the other hand, when the number of polymer chains becomes sufficiently large (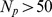) the response is increasingly well approximated by relatively smooth force-extension curves, similar to the cohesive laws routinely used in fracture mechanics [36]–[37].

We identify two distinct regimes in the force-extension plots shown in Fig 5. For small displacements, the force peaks tend to increase in magnitude as the extension increases. This is the displacement-strengthening regime which is expected to play a significant role in resisting crack initiation and controlling stable crack growth. On the other hand, for large displacements the force peaks tend to decrease with increasing extension corresponding to failure of end bonds and the detachment of polymer chains. This is the displacement-weakening regime which is expected to allow for the growth of instabilities and dynamic crack propagation. This displacement weakening instability is a collective effect of the polymer network and is qualitatively different from the continuing upward trend observed for a single polymer in Fig. 3(b) and 3(c).

The magnitude of the force drops in Fig. 5(b) relative to the overall force scale is large compared to the force fluctuations in Fig. 5(c) where the curves are much smoother. While small scale roughness persists in Fig. 5(c), these irregularities are in most cases of the order of 5% of the maximum force.

Another important difference is the variability in size of the force drops of significantly in Fig. 5(b) compared with Fig. 5(c) or Fig 3(b) and 3(c). The force drops of smaller magnitude correspond in most cases to the breakage of individual sacrificial bonds and unfolding of the hidden loops, whereas the force drops of larger magnitude typically represent detachment of polymers from the backbone. This is because the breakage of an internal sacrificial bond leads to the unfolding of a single hidden loop. This only reduces the stiffness of the system slightly and causes a small force drop. However, once the polymer chain detaches, the force supported by it drops to zero. The force drop in this case is usually bigger especially if the chain is close to its maximum extension.

The detachment of the polymer chain can occur either because the bond connecting it to the fibril breaks (weak end bond) or because the force in the polymer chain exceeds the polymer strength. While polymers also detach from the backbone in Fig. 5(c), the number of polymers is large enough that detachment of single polymer does not significantly impact the system as a whole.

If the number of polymers contributing to the polymeric gel system is small, the accuracy of large-scale models of fracture in mineralized collagen fibrils may depend critically on modeling these discrete effects characteristic of the force-extension curves. However, if the number of polymers is sufficiently large, the interfacial description may be greatly simplified. Our results suggest that the quasi-static cohesive law in this case may be represented by a smooth curve parameterized by three variables: (i) the magnitude of the maximum force that the interface can resist (*F*_max_), (ii) the magnitude of the maximum displacement at complete separation (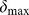) and (iii) the displacement corresponding to 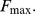 The self-similarity of the force extension curves (in the limit of large number of polymers) enabled such parameterization. We have found that an expression of the form 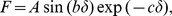 fits the results fairly well (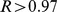). In this expression
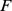
and 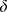 are the force and displacement normalized by their maximum values respectively. 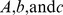 are fitting constants. For a good approximation: 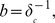 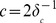 and 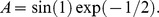 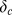 is the normalized displacement at maximum force.

**Figure 6:**
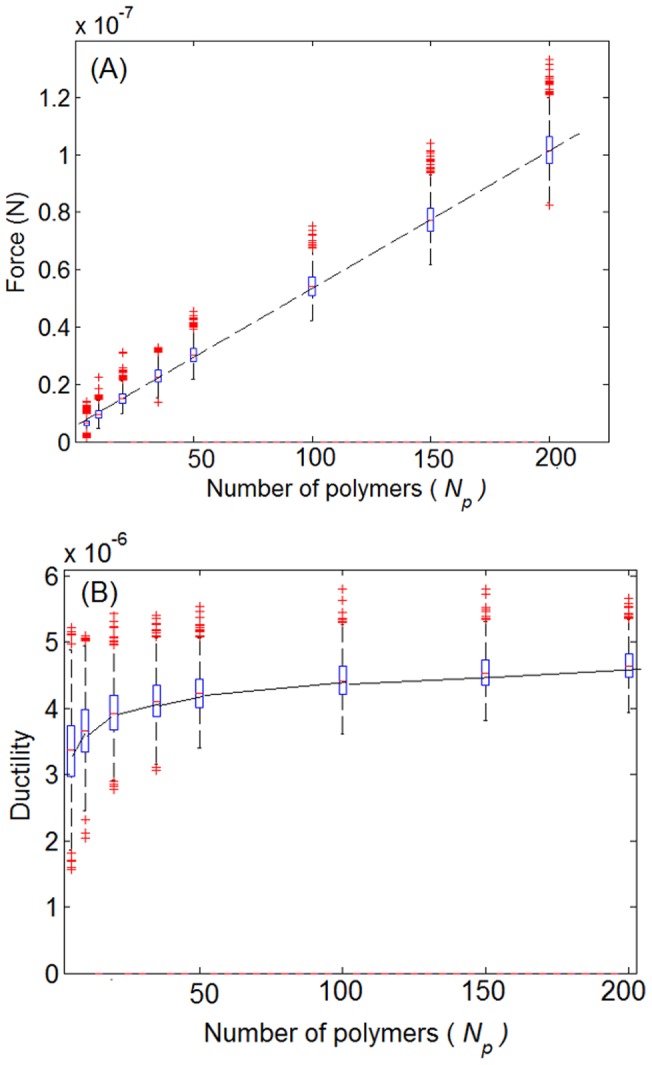
The effect of the number of polymers (*N_p_*) on the maximum force and ductility (maximum extension) of the polymer group. On each box, the central mark is the median, the edges of the box are the 25th and 75th percentiles, the whiskers extend to the most extreme data points not considered outliers, and outliers are plotted individually. (a) The maximum force increases linearly with increasing the number of polymers and (b) the maximum extension is weakly dependent on the number of polymers.

Examination of the trends in Figs. 5(b) and 5(c) suggest that the maximum force *F_max_* is proportional to the number of polymers while the maximum displacement 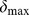 is weakly dependent on the number of polymers. This is verified quantitatively in Fig. 6 which illustrates statistical results for these parameters as a function of the number of polymers *N_p_*. For each value of *N_p_* the numerical experiment was repeated for 1500 realizations. The distribution of the maximum force, for a fixed value of *N_p_* , is non-Gaussian. It can be represented, however, by a finite sum of Gaussian functions. On the other hand, the maximum extension distribution was found to be approximately Gaussian.

The increase in the maximum force with the increase in the number of polymers follows directly from the parallel arrangement of the polymers. At any given displacement 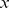 the total force supported by the system is given by:
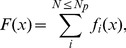
(7)where
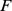
is the total force, 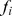
is the force in the *i* ^th^ polymer chain computed by Eqn.(3) and 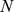
is the number of unbroken polymer chains at displacement *x*. In the displacement strengthening regime,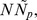 so that the larger
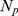
the higher the maximum force 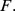


On the other hand, the approximate independence of the maximum extension on the number of polymers follows from the fact that the maximum displacement depends only on the contour length of the last surviving polymer chain. This is taken to be a random variable independent of the number of polymers.

Finally we examine the effect of the strength of the end bonds on the macroscopic properties of the polymeric system. End bonds may have different structure from internal bonds and hence the two groups might differ in their strength. The distinction is then important as it impacts the dynamics of the SBHL and could have potential therapeutic implications.

Following the discussion in Section III, if the strength of the end bonds is less than the polymer chain strength, the bonds will break and the polymers will prematurely detach before the SBHL system is fully utilized. This lowers both the strength and toughness of the system. On the other hand, if the end bonds are stronger than the polymer, all the internal sacrificial bonds in each chain will break before detachment and each polymer will extend to its fullest. This, in turn, enables maximal contribution of each polymer to the overall system strength and toughness.

**Figure 7:**
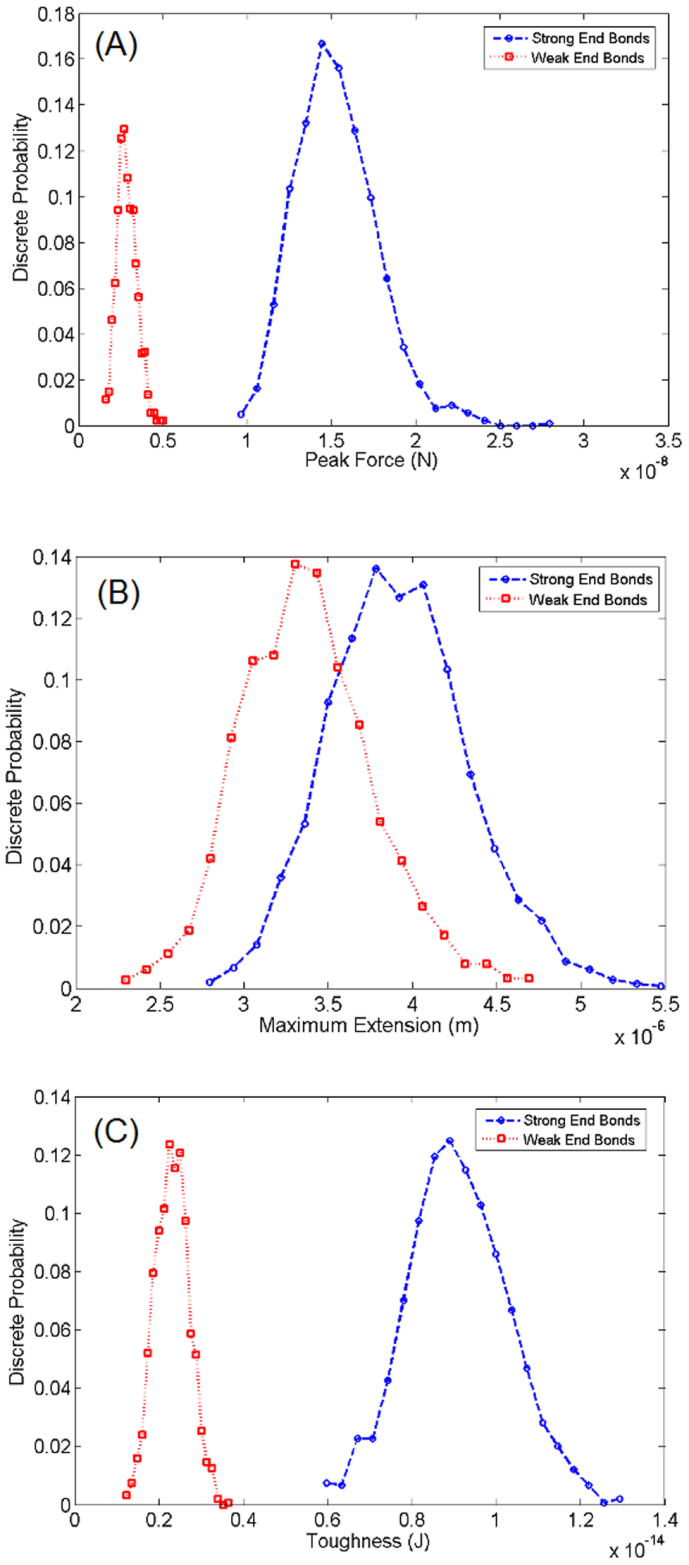
Effect of the strength of the bonds connecting the polymer chains to the mineralized collagen fibrils. Polymers with strong end bonds (blue circles) lead systematically to (a) higher strength, (b) ductility, and (c) toughness, compared to cases where the end bonds are weaker than the polymer itself (red squares). [Depicted probabilities are discrete].

We investigated the two scenarios of weak and strong end bonds by simulating 1500 realizations of polymers with the SBHL system. In both cases, the geometric properties of the polymer chains, such as the contour length and the length of the hidden loops, are drawn from the same random distribution. In one group of simulations, the strength of the end bonds is chosen from the same probability distribution governing the bonds. In this case, the strength of both types of bonds is drawn from a uniform distribution of force thresholds ranging from a minimum value of 1pN to a maximum value of 1nN. In another group of simulations, the strength of the end bonds is chosen greater than the strength of the polymer chain (> 1nN) so that only when the polymer is fully extended it detaches by breaking internally. The results of the numerical investigation are shown in Fig. 7. The cases with strong end bonds (circles) attain on average higher maximum forces, longer extensions and larger toughness values than the cases with nominal (weak) end bonds (squares).

By preventing premature detachment of the polymer chains, the mechanical performance of the polymer group, as measured by strength, ductility and toughness, is significantly enhanced. This suggests that development of therapies which target enhancement of end bonds may play an important role in reducing bone health problems, especially in the elderly.

## Conclusion

Problems involving dynamics of cohesively held interfaces arise broadly in biological [Martin *et al.*, 1998] as well as in Engineering [36], [38] and geophysical [39]–[40] applications. Common to all of these applications are fundamental physical processes involving deformation, rupture nucleation, propagation and arrest. In strongly nonlinear problems, like dynamic fracture, small scale instabilities can lead to large scale system fragilities [1], [41]–[43] and it is imperative to understand how the microscopic processes influence the crack macroscopic response. In the case of bone, the internal interfaces between the mineralized collagen fibrils may fail under different loading conditions, and the details of the resulting dynamic rupture can determine whether only a part or the whole of the body part (e.g. knee) will fracture.

The separation of the mineralized collagen fibrils under shear or tension is resisted by a special type of polymeric glue that is composed of polymers with sacrificial bonds and hidden length (SBHL) system. The constitutive response of this glue controls the strength and ductility of the fibril plates.

Several mathematical models have been proposed to explain polymer nonlinear elasticity. Of these models, the worm like chain (WLC) and the freely jointed chain (FJC) models are the most widely used [13]–[15], [36]. The two models differ in their idealization of the polymer chain and their prediction of the force extension response.

In this paper we investigated the mechanics and implications of entropy-based energy dissipation in polymer chains with SBHL system using the worm like chain model. Our primary focus was on the influence of the SBHL system on the larger scale mechanical observables such as toughness, ductility and mescosopic constitutive response.

Our numerical results indicate that the SBHL system generally enhances the toughness and the strength of the polymeric system and this should allow for better resistance to crack initiation and propagation as well as better performance under dynamic loading. These properties can be used in designing base isolators to damp vibrations in structures subjected to ground motions [44].

Investigation of the dependence of polymer toughness and ductility on the internal variables of the SBHL system reveals constraints on the optimum number of sacrificial bonds as well as the strength of the bonds in order for the SBHL system to be fully utilized. In particular, the net gain in toughness may not increase in the presence of a larger number of bonds. Moreover, we show that the presence of strong internal sacrificial bonds can reduce the polymer ductility and toughness. Unlike the strength requirements for the internal bonds, strong end bonds are required to prevent premature detachment of the polymer chain and to allow the SBHL system to fully operate. These observations must be taken into account in developing therapeutic procedures that aim to enhance bone mechanical behavior or in designing bio-inspired composites with improved fracture resistance properties.

We also investigated the collective behavior of a system of parallel polymers constrained by the same displacement controlled loading. In real elastomeric networks, polymers can develop crosslinks with each other. This crosslinking will definitely affect the force distribution and ductility in the polymeric system [45]. The study of the parallel setup, however, acts as a starting point for understanding the constitutive response at larger scales. It is also relevant to a wide range of applications such as modeling diatoms [27], and parallel polyprotein dimers [28]. Moreover, it serves as a good approximation for the glue between the bone fibrils in young healthy individuals. Aging increases the crosslinking between polymers and reduces their toughness and ductility. This effect will be investigated further in our future work.

For a small number of polymer chains, we show that the cohesive law describing the force- extension response carries a clear signature of the microscopic processes of bond breakage and polymer detachment. This is manifested in the existence of force drops of magnitude comparable to the magnitude of the maximum force supported by the system. The implementation of these details in any large scale model of fracture is required because they can significantly affect crack initiation and propagation. We also showed that these details agree with observations from AFM experiments done on dentin molecules [35].

For a large number of polymer chains, however, the force fluctuations due to these microscopic processes are small compared to the amplitude of the maximum forces achieved in the system. The cohesive law in this case is greatly simplified and can be approximated by a smooth curve characterized by two numbers: the maximum force and the maximum extension.

Our results show that the maximum force supported by the polymeric system is proportional to the polymer density (number of polymer chains per unit area) and so is the system toughness. These results are relevant for understanding changes in the bone mechanical response with aging. Since the number of polymers produced by the osteocytes may decrease as the individual ages, this investigation reveals possible mechanisms for bone toughness degradation with age other than loss of bone density. It also suggests that therapeutic interventions that stimulate the production of the polymeric gel can help enhance bone health especially in the elderly [9], [20].

A future extension of this study will involve use of the derived cohesive law as a boundary condition for individual elements in large scale dynamical models of bone hierarchal structure. These models will ultimately incorporate the structural complexity of the human bone at different scales to determine the combined effects of microscopic mechanisms of the SBHL system with material and geometric heterogeneities in resisting fracture. Multiscale modeling is a critical ingredient in understanding the fracture toughness in bone as well as in designing new composite materials with high levels of strength and toughness.

## Supporting Information

File S1 **Supporting Information Part A and Figure** **S1.** Representative Force extension data (dots) from AFM experiments on bone glue. Different colors correspond to retraction curves in Calcium ion solutions after different delay [Source: Thompson et al., 2001]. The data is fitted by the worm like chain model (smooth curves). [Force is normalized by 70 pN and extension is normalized by 2.35 nm].
(DOCX)

File S2
**Supporting Information Part B and Figure** **S2.** Force-extension plots of a polymer chain (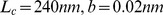) with different numbers of sacrificial bonds *n.*
(DOCX)
