# Supplementary material for: Dynamics of Polymer Molecules with Sacrificial Bond and Hidden Length Systems: Towards a Physically-Based Mesoscopic Constitutive Law
Source: PLoS One. 2013 Apr 2;8(4):e56118. doi: 10.1371/journal.pone.0056118 (PMC3614968; doi:10.1371/journal.pone.0056118)

# SUPPORTING INFORMATION PART A

The Worm like chain model idealizes the polymer chain as a continuously flexible rod and accounts for the bending stiffness of individual monomers [13-14, 30]. Figure A1 shows that the WLC model provides a good fit for the force extension curves observed in AFM experiments on bone glue in the limit of large extensions. The data points were obtained by digitizing Fig. 1 in Hansma et al., 2005. The accuracy of the digitization process was limited by the quality of the printed plots. Nonetheless, in the limit of large displacements the accuracy is improved. The MATLAB curve fitting toolbox was used to estimate the fitting parameters automatically.

Different data sets in Fig. S1 correspond to experiments with different initial conditions. Polymers are left in Calcium ion solutions for 1, 10 and 100 sec before being pulled and then left to retract [11, 33]. All fits yield indistinguishable values for the contour length but different values of the persistence length and have R-square values exceeding 0.97. The persistence length is small (of the order of 0.02nm). This suggests that more than one single polymer are pulled simultaneously in these experiments. It is also possible that crosslinks are developed between individual polymers during the delay time and this also affects the value of the apparent persistence length (that is inferred based on a single polymer assumption).

**Figure S1.** **Representative Force extension data (dots) from AFM experiments on bone glue**. Different colors correspond to retraction curves in Calcium ion solutions after different delay [Source: Thompson et al., 2001]. The data is fitted by the worm like chain model (smooth curves). [Force is normalized by 70 pN and extension is normalized by 2.35 nm.]


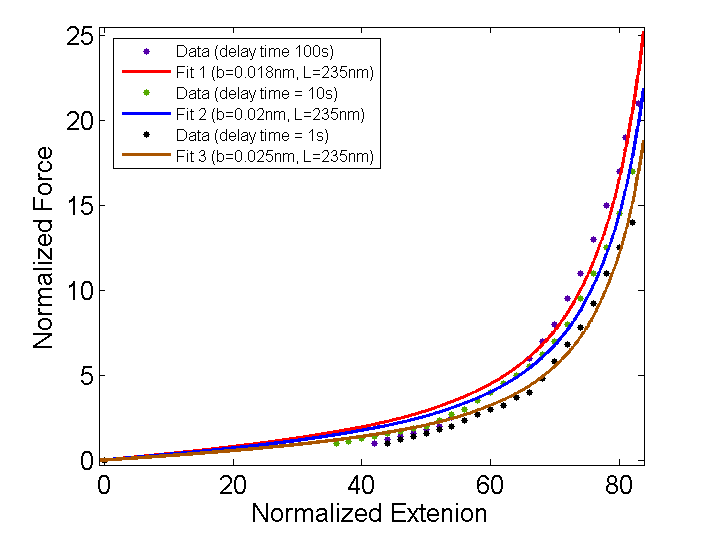

Supplement: File S1 — Supporting Information Part A and Figure S1. Representative Force extension data (dots) from AFM experiments on bone glue. Different colors correspond to retraction curves in Calcium ion solutions after different delay [Source: Thompson et al., 2001]. The data is fitted by the worm like chain model (smooth curves). [Force is normalized by 70 pN and extension is normalized by 2.35 nm]. (DOCX) [file pone.0056118.s001.docx]
