# Supplementary material for: Dynamics of Polymer Molecules with Sacrificial Bond and Hidden Length Systems: Towards a Physically-Based Mesoscopic Constitutive Law
Source: PLoS One. 2013 Apr 2;8(4):e56118. doi: 10.1371/journal.pone.0056118 (PMC3614968; doi:10.1371/journal.pone.0056118)

# SUPPORTING INFORMATION PART B

The force-extension law for a polymer chain with the SBHL system is given by Eqn. (3) in the text. This constitutive description is an example of a state variable formulation in which the state variable is the number of unbroken bonds *n*. The force *f* supported by the chain depends on the available length *L_a_*(Eqn. (3)) which in turn depends on the number of unbroken sacrificial bonds *n* (Eqn. (2)). By substituting Eqn. (2) into Eqn. (3) we arrive at:

$f=\frac{k_{B}T}{b}\left[\frac{1}{4}\left(1-\frac{x}{L_{c}-\sum_{i=1}^{n} l_{i}}\right)^{-2}-\frac{1}{4}+\frac{x}{L_{c}-\sum_{i=1}^{n} l_{i}}\right].$ (SB1)

The number of unbroken sacrificial bonds *n* is updated by a threshold fracture criterion. Whenever the chain force *f* exceeds the strength of a bond, this bond breaks reducing *n* in Eqn. (B1) by unity.

In this Supporting Information Part we investigate the dependence of the force *f* on the number of bonds *n* in the limit$n\to\infty$. To make the problem tractable analytically, we assume all the hidden loops are of equal length$l=L_{c}/\left(2n+1\right)$. The available length is then given by$L_{a}=\left[\left(n+1\right)/(2n+1)\right]L_{c}$.

We consider the continuum version of Eqn. (B1). Replacing $L_{c}-\sum_{i=1}^{n} l_{i}$with $L_{a}$and differentiating Eqn. (A1) with respect to *n*, we arrive at:

$\begin{matrix} \frac{\partial f}{\partial n}=-\frac{k_{B}T\left(dL_{a}/dn\right)}{b}\frac{x}{L_{a}^{2}}\left[\frac{1}{2}\left(1-\frac{x}{L_{a}}\right)^{-3}+1\right], \\ \text{ } \end{matrix}$ (SB2)

and

$\frac{dL_{a}}{dn}=-\frac{(2n+1)L_{c}-2nL_{c}}{(2n+1)^{2}}=-\frac{L_{c}}{(2n+1)^{2}}$. (SB3)

Let $\varepsilon\to0$and $n\sim O\left(\frac{1}{\varepsilon}\right)$ then

$L_{a}\to\frac{L_{c}}{2}\text{ and }\frac{dL_{a}}{dn}\to0.$ (SB4)

That is, in the limit of large number of sacrificial bonds, the available length is approximately one half of the contour length and is insensitive to bond breakage. Moreover, from Eqn. (B2) we obtain:

$\frac{\partial f}{\partial n}\sim\left(\frac{\varepsilon}{2+\varepsilon}\right)^{2}\to0$. (SB5)

That is, the force in the chain varies as $1/n$when *n* is large. This implies that the force-extension curves for polymers with a large number of sacrificial bonds vary weakly with the variation of the number of sacrificial bonds. This is shown in Fig.B1 which illustrates the constitutive response of a polymer chain with different numbers of sacrificial bonds. The curves are indistinguishable from each other except for large extensions when the number of unbroken sacrificial bonds becomes small and discrete effects in individual realizations become pronounced. Since toughness is measured by the area under the force-extension curve, there is little or no change in toughness as the number of sacrificial bonds becomes large. This explains the asymptotic behavior for the curves in Fig. 4 in the text.

**Figure S2. Force-extension plots of a polymer chain ($L_{c}=240nm$,$b=0.02nm$) with different numbers of sacrificial bonds *n.***


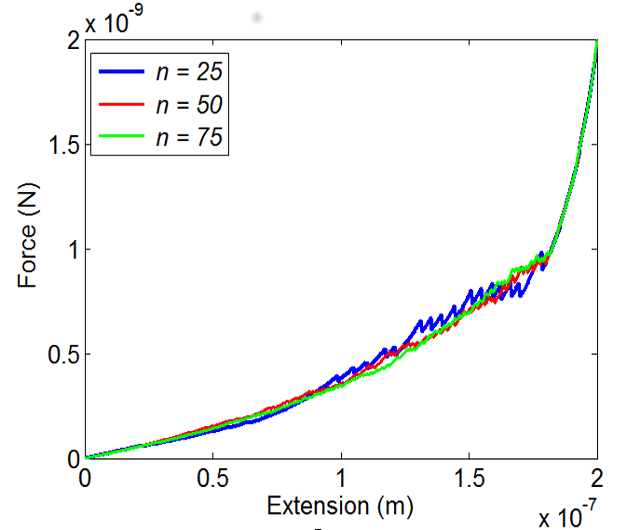

Supplement: File S2 — Supporting Information Part B and Figure S2. Force-extension plots of a polymer chain () with different numbers of sacrificial bonds n. (DOCX) [file pone.0056118.s002.docx]
